# Out-of-Hospital Cardiac Arrest Before, During, and After the Pandemic of COVID-19

**DOI:** 10.3390/jcm15166229

**Published:** 2026-08-12

**Authors:** Goran Rakic, Aleksandar Djuricin, Nikolina Maric, Mirka Lukic Sarkanović, Maja Stefanovic, Biljana Draskovic, Srdjan Gavrilovic, Milena Joksic Zelic, Velibor Vasovic, Radojka Joksic-Mazinjanin

**Affiliations:** 1 Medical Faculty, University of Novi Sad, 21000 Novi Sad, Serbia; goran.rakic@mf.uns.ac.rs (G.R.); aleksandar.djuricin@mf.uns.ac.rs (A.D.); mirka.lukic-sarkanovic@mf.uns.ac.rs (M.L.S.); maja.stefanovic@mf.uns.ac.rs (M.S.); biljana.draskovic@mf.uns.ac.rs (B.D.); srdjan.gavrilovic@mf.uns.ac.rs (S.G.); velibor.vasovic@mf.uns.ac.rs (V.V.); radojka.joksic-mazinjanin@mf.uns.ac.rs (R.J.-M.); 2 Pediatric Surgery Clinic, Institute for Child and Youth Health Care Vojvodina, 21000 Novi Sad, Serbia; 3 Institute for Emergency Medical Services, 21000 Novi Sad, Serbia; 4 University Clinical Center of Vojvodina, 21000 Novi Sad, Serbia; 5 Institute for Cardiovascular Diseases of Vojvodina, 21204 Sremska Kamenica, Serbia; 6 Institute for Pulmonary Diseases of Vojvodina, 21204 Sremska Kamenica, Serbia; 7 Department of Emergency Medicine, Health Center Becej, 21220 Becej, Serbia; milenajoksiczelic@gmail.com

**Keywords:** coronavirus disease 2019, out-of-hospital cardiac arrest, cardiopulmonary resuscitation, return of spontaneous circulation, mortality

## Abstract

**Background/Objectives:** This study aimed to evaluate the incidence and outcomes of emergency medical service (EMS)-treated out-of-hospital cardiac arrest (OHCA) and to determine whether predictors of outcomes differed across the pre-pandemic, pandemic, and post-pandemic periods. **Methods:** A retrospective observational study was conducted over a six-year period and included 1150 patients with EMS-treated OHCA. Patients were categorized into three groups according to the study period. **Results:** The incidence of EMS-treated OHCA differed significantly across the three study periods (χ^2^ = 15.184, *p* = 0.001), with the highest number of cases observed during the pandemic. The rate of return of spontaneous circulation (ROSC) also varied significantly between periods (*p* = 0.035). Although the highest overall mortality was observed during the pandemic period (97.5%), differences in overall mortality across the study periods did not reach statistical significance (*p* = 0.079). Variables independently associated with ROSC were EMS response time, initial cardiac rhythm, and the administration of adrenaline and atropine. Age and initial cardiac rhythm were independently associated with mortality. No significant interactions were observed between study period and the identified predictors of ROSC or mortality. **Conclusions:** The COVID-19 pandemic was associated with a significant increase in EMS-treated OHCA incidence and poorer patient outcomes, including lower ROSC and survival rates. Although outcomes improved in the post-pandemic period, multivariable analysis demonstrated that the pandemic period itself was not independently associated with ROSC or mortality after adjustment for relevant clinical factors. Instead, outcome differences were primarily explained by established clinical predictors, including patient age, initial cardiac rhythm, EMS response time, and resuscitation-related factors. Prospective studies incorporating more detailed data on patient characteristics, the quality of resuscitation, and organizational characteristics of the healthcare system may enable more accurate outcome modeling.

## 1. Introduction

Coronavirus disease 2019 (COVID-19) emerged in December 2019 and rapidly developed into a global public health emergency. On 11 March 2020, the World Health Organization (WHO) officially declared COVID-19 a pandemic [1].

During the early stages of the pandemic, healthcare systems were unprepared to cope with the unprecedented increase in patient volume. The majority of healthcare resources were redirected toward the management of patients with COVID-19, resulting in deterioration of healthcare delivery and poorer outcomes among patients with other acute medical conditions. Increased incidence and mortality were observed among patients with acute coronary syndromes and acute ischemic stroke. Furthermore, delays in seeking medical attention and postponement of surgical procedures due to limited hospital capacity and workforce shortages led to an increase in complications among patients with acute abdominal conditions [2,3,4].

The incidence of out-of-hospital cardiac arrest (OHCA) also increased substantially during the COVID-19 pandemic. Recommendations for social distancing and restrictions on social interactions were associated with changes in healthcare-seeking behavior and emergency care utilization. This was reflected in delays in contacting emergency medical services (EMS) and in seeking emergency care after symptom onset. Simultaneously, delayed initiation of basic life support due to the absence of bystanders resulted in lower rates of return of spontaneous circulation (ROSC) and reduced patient survival [5,6].

Observational studies and meta-analyses investigating the incidence, epidemiological characteristics, and outcomes of OHCA during the COVID-19 pandemic, compared with the pre-pandemic period, demonstrated reductions in the proportion of shockable rhythms, the use of automated external defibrillators, and endotracheal intubation. In contrast, EMS response times, the proportion of cardiac arrests occurring at home, and the use of supraglottic airway devices increased. In addition, rates of prehospital ROSC, survival to hospital admission, and survival to hospital discharge decreased significantly, while neurological outcomes among survivors of OHCA were worse during the pandemic period [7,8].

Although the effects of the COVID-19 pandemic on OHCA have been well documented, it remains unclear whether these changes were transient or persisted into the post-pandemic period. Furthermore, it is unknown whether the prognostic significance of individual predictors of OHCA outcomes differs between the pre-pandemic, pandemic, and post-pandemic periods. Initially, following the onset of the COVID-19 pandemic, numerous studies examined its impact on out-of-hospital cardiac arrest. However, its effects during the post-pandemic period have received limited attention. Evaluating this period is essential for assessing the recovery of healthcare systems in this critical area of patient care. Ruiz Azpiazu et al. reported that survival among patients with OHCA improved following the COVID-19 pandemic, accompanied by better neurological outcomes at hospital discharge [9].

Therefore, the present study aimed to evaluate the incidence and outcomes of EMS-treated OHCA and determine whether the effects of key outcome predictors differed across the pre-pandemic, pandemic, and post-pandemic periods.

## 2. Materials and Methods

### 2.1. Study Design and Settings

A retrospective observational study was conducted at the Emergency Medical Service Novi Sad (EMS Novi Sad), the University Clinical Centre of Vojvodina (UCCV), the Institute for Pulmonary Diseases of Vojvodina (IPDV), and the Institute of Cardiovascular Diseases of Vojvodina (ICDV). The study covered a six-year period from 15 March 2018, to 14 March 2024.

The study included patients who experienced OHCA during the specified study period and for whom advanced life support (ALS) was initiated by EMS Novi Sad. All OHCA cases included in the analysis were witnessed either by a bystander or by EMS personnel.

In the Republic of Serbia, healthcare is predominantly delivered through a public system financed by the National Health Insurance Fund. Insured individuals receive healthcare services within this system without direct payment, while emergency medical care is provided irrespective of health insurance status. The public healthcare system is organized into three levels: primary care, comprising primary healthcare centers and EMS; secondary care, provided by general hospitals; and tertiary care, delivered by specialized institutes and university clinical centers. The present study was conducted in an area without secondary-level healthcare facilities; therefore, patients requiring hospital care are referred directly from primary- to tertiary-level institutions.

### 2.2. Data Sources

EMS Novi Sad provides prehospital emergency medical care for a population of approximately 370.000 inhabitants. The population of the EMS catchment area increased by approximately 2000 inhabitants annually, from approximately 367,000 in 2018 to 377,000 in 2023. The service operates a dispatch center responsible for call reception and triage, as well as nine emergency medical teams per shift. Each team consists of an emergency medicine specialist or a physician, an emergency medical technician, and a driver trained in basic life support.

All emergency medical teams are equipped uniformly and possess the necessary equipment for ALS. Each team is equipped with a manual biphasic defibrillator, infraglottic airway devices (supraglottic airway devices were used only in a limited number of cases), a video laryngoscope, and all medications required for the provision of ALS according to established protocols. Emergency calls are recorded within the dispatch center, while patient assessment findings, therapeutic interventions, stabilization measures, and EMS response times are documented in the EMS medical records.

For patients in whom ROSC was achieved following ALS, additional data were obtained from hospital databases of the UCCV, IPDV, or ICDV, depending on the institution to which the patient was transported after resuscitation.

Prehospital data were extracted from electronic dispatch records and EMS medical documentation. The following variables were collected:

- Demographic characteristics of patients with cardiac arrest (sex and age);
- Presence of a witness to the cardiac arrest;
- EMS response time, defined as the interval between the emergency call and arrival of the EMS team at the scene. In cases where the EMS team directly witnessed the cardiac arrest, a response time of zero minutes was assigned and excluded from the calculation of the overall mean response time;
- Whether cardiopulmonary resuscitation (CPR) was initiated immediately by a bystander or an EMS team;
- ALS interventions performed by EMS personnel;
- Achievement of ROSC and survival to hospital admission.

Regarding ALS interventions performed by EMS teams, the following variables were collected:

- Airway management technique (endotracheal intubation, laryngeal mask airway, or bag-valve-mask ventilation);
- Administration of adrenaline and the number of administered ampoules;
- Administration of atropine;
- Administration of amiodarone;
- Administration of isotonic crystalloid solutions;
- Defibrillation and the number of delivered shocks.

Atropine has not been recommended for the management of OHCA in ALS guidelines since 2010. Nevertheless, its use has persisted at our institution and in several other EMS systems in Serbia under narrowly defined circumstances. It may be considered in younger patients without substantial comorbidities who exhibit persistent pulseless electrical activity despite high-quality chest compressions, repeated administration of adrenaline at three-minute intervals, and the identification and management of potentially reversible causes of cardiac arrest. Because transport during ongoing CPR is not feasible within our EMS system, local institutional practice permits atropine administration as an additional therapeutic measure in these highly selected cases.

For patients who achieved ROSC, additional data were collected from hospital medical records of the ICDV, IPDV, and UCCV. Hospital records were reviewed to obtain information regarding survival to hospital discharge, neurological status at discharge, mortality within the first 24 h following admission, and mortality occurring from the second hospital day until discharge. Overall mortality was defined as death occurring at any time from OHCA until hospital discharge.

Neurological outcomes at hospital discharge were assessed using the Cerebral Performance Category (CPC) scale. CPC scores were assigned by the attending physician and documented in the discharge summary or medical progress notes. When a CPC score was not explicitly documented, it was determined retrospectively based on the description of the patient’s neurological status at discharge and the corresponding Glasgow Coma Scale (GCS) score recorded in the medical documentation.

### 2.3. Study Population

The study encompassed a six-year period, and participants were categorized into three groups according to the period during which cardiac arrest occurred.

The first period represented the pre-pandemic period and included the two years preceding the COVID-19 pandemic. The second period corresponded to the COVID-19 pandemic and began on 15 March 2020, when a state of emergency and nationwide lockdown were implemented in the Republic of Serbia. For the purposes of this study, the pandemic period was defined as ending on 14 March 2022, by which time more than 50% of the population had been vaccinated and the numbers of newly diagnosed COVID-19 cases, hospitalizations, and deaths had substantially declined. At the same time, COVID-19 passes and restrictions on the size of indoor gatherings were lifted. The third period represented the post-pandemic period.

**Figure 1:**
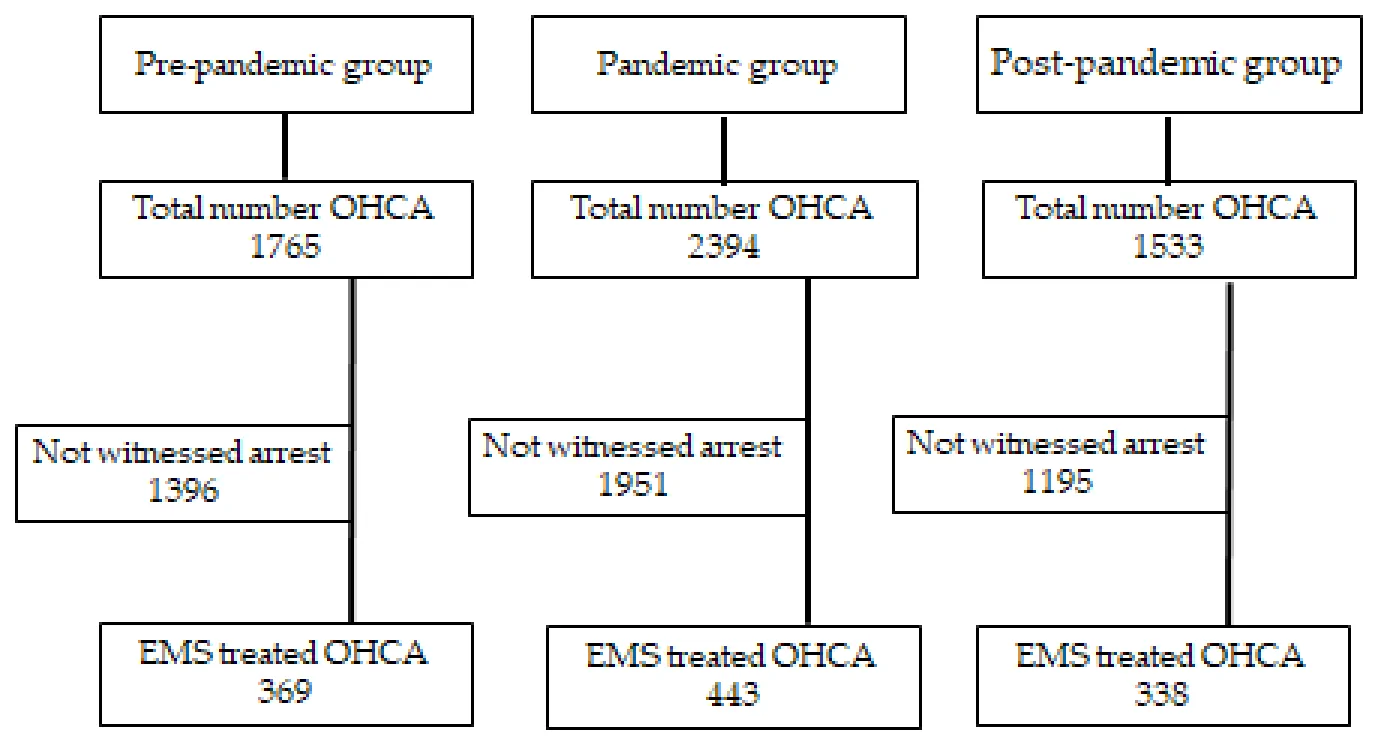
Distribution of patients according to the study period.

Accordingly, patients were assigned to one of three study groups:

- Pre-pandemic group: patients who experienced EMS-treated OHCA between 15 March 2018, and 14 March 2020;
- Pandemic group: patients who experienced EMS-treated OHCA between 15 March 2020, and 14 March 2022;
- Post-pandemic group: patients who experienced EMS-treated OHCA between 15 March 2022, and 14 March 2024 (Figure 1).

Patients were eligible for inclusion if EMS physicians initiated ALS for cardiac arrest of any etiology in the prehospital setting. Patients with sudden cardiac arrest in whom ALS was not initiated by EMS personnel were excluded.

### 2.4. Statistical Analysis

Data were presented as mean ± standard deviation (SD) or median with interquartile range (IQR; Q1–Q3), as appropriate for the distribution of the data. Categorical variables were expressed as frequencies and percentages (N, %).

Normality of data distribution was assessed using the Kolmogorov–Smirnov test. Comparisons among the three study periods (pre-pandemic, pandemic, and post-pandemic) were performed using one-way analysis of variance (ANOVA) or the Kruskal–Wallis test for continuous variables, followed by independent-samples *t*-tests or Mann–Whitney U tests for post hoc comparisons, respectively, with Bonferroni correction for multiple comparisons. As neither the ANOVA nor the Kruskal–Wallis test yielded statistically significant results, post hoc analyses were not performed.

Categorical variables were compared using the chi-square test with Bonferroni correction. Fisher’s exact test or Monte Carlo simulation was applied when appropriate. Relative risks (RRs), odds ratios (ORs), 95% confidence intervals (95% CIs), and corresponding *p*-values were calculated for ROSC, survival to hospital discharge, and overall mortality. Predictors of ROSC and mortality were evaluated using univariable and multivariable logistic regression analyses, including interaction terms with the study period. Variables that demonstrated statistical significance in the univariable analyses were included in the multivariable models. The main effect of the study period (pre-pandemic, pandemic, and post-pandemic), as well as interaction terms between the study period and selected predictors, was evaluated to determine whether the pandemic period modified the associations between individual predictors and the outcomes. Before constructing the multivariable models, collinearity among the independent variables was assessed by examining the correlation matrix. As no significant correlations indicative of multicollinearity were identified, all selected variables were retained in the multivariable analyses. Model performance was assessed using the Hosmer–Lemeshow goodness-of-fit test and pseudo-R^2^ measures (Cox–Snell and Nagelkerke).

Statistical significance was defined as *p* < 0.05. All analyses were performed using IBM SPSS Statistics version 23. Results are presented in tabular form.

## 3. Results

### 3.1. Demographic Characteristics and Immediate CPR Attempt

A total of 1150 patients who experienced EMS treated OHCA- during the six-year study period were included in the analysis. The annual incidence of OHCA for which EMS teams initiated resuscitation was 50.1 per 100,000 inhabitants during the pre-pandemic period. It increased to 59.5 per 100,000 inhabitants during the COVID-19 pandemic and subsequently decreased to 44.9 per 100,000 inhabitants in the post-pandemic period, representing the lowest annual incidence observed across the study periods.

The incidence of EMS treated OHCA differed significantly among the study periods (χ^2^ = 15.184, df = 2, *p* = 0.001). The highest incidence was recorded during the pandemic period, while the lowest incidence was observed in the post-pandemic period. Compared with the pre-pandemic period, the pandemic period was associated with a significantly higher incidence of EMS treated OHCA (RR = 1.20, 95% CI 1.07–1.34; OR = 1.27, 95% CI 1.10–1.46; *p* = 0.004). Similarly, the incidence of EMS treated OHCA was significantly higher during the pandemic than during the post-pandemic period (RR = 1.31, 95% CI 1.15–1.49; OR = 1.39, 95% CI 1.16–1.66; *p* < 0.001). In contrast, no significant difference was observed between the post-pandemic and pre-pandemic periods (RR = 0.92, 95% CI 0.80–1.05; OR = 0.90, 95% CI 0.74–1.10; *p* = 0.330).

Accordingly, the risk of EMS treated OHCA was approximately 20% higher during the pandemic than in the pre-pandemic period and approximately 31% higher than in the post-pandemic period. The study groups were comparable with respect to sex, age, EMS response time, immediate initiation of resuscitation, and bystander CPR rates (all *p* > 0.05) (Table 1). No cases involved the use of an automated external defibrillator (AED).

### 3.2. Initial Cardiac Rhythm and Advanced Life Support Interventions

Across all three study periods, the majority of patients had a non-shockable initial rhythm, most commonly asystole, with no significant differences in the proportion of patients presenting with asystole between the three study periods. (50.1% vs. 55.3% vs. 52.4%, respectively; *p* = 0.334).

Airway management strategies differed significantly across the study periods (*p* < 0.001). Post hoc analysis with Bonferroni correction demonstrated that endotracheal intubation was performed significantly less frequently during the pandemic than during both the pre-pandemic and post-pandemic periods (both *p* < 0.001). Conversely, bag-valve-mask ventilation was used significantly more frequently during and after the pandemic compared with the pre-pandemic period (both *p* < 0.001).

Although the proportion of patients receiving atropine did not differ significantly among the study periods (*p* = 0.061), but the number of atropine ampoules administered differed significantly (*p* = 0.016), with greater use observed during the pandemic than in the pre-pandemic period (*p* = 0.004). Amiodarone administration also differed significantly among the study periods (*p* = 0.005), being more frequent before the pandemic than during (*p* = 0.006) or after the pandemic (*p* = 0.003).

The use of isotonic crystalloid solutions differed significantly among the study periods (*p* = 0.005) and was lower during the pandemic compared with both the pre-pandemic and post-pandemic periods (both *p* < 0.01) (Table 2).

### 3.3. Comparative Analysis of OHCA Care Outcomes

Rates of ROSC differed significantly among the study periods (χ^2^ = 6.693, df = 2, *p* = 0.035), with the lowest ROSC rate observed during the pandemic. Compared with the pandemic period, the likelihood of achieving ROSC was approximately 1.6-fold higher during both the pre-pandemic period (RR = 1.47, 95% CI 1.03–2.09; OR = 1.56, 95% CI 1.04–2.34; *p* = 0.030) and the post-pandemic period (RR = 1.52, 95% CI 1.06–2.16; OR = 1.65, 95% CI 1.08–2.43; *p* = 0.019).

Prehospital mortality was higher during the pandemic than during the pre-pandemic and post-pandemic periods. Differences in patient outcomes between the study periods were observed at successive stages of care.

Prehospital mortality was highest during the pandemic, whereas comparable rates were observed in the pre-pandemic and post-pandemic periods. Mortality within the first 24 h after hospital admission did not differ significantly among the study periods (*p* = 0.908). Mortality from the second hospital day until discharge was highest during the pandemic (57.1%), compared with the pre-pandemic (44.4%) and post-pandemic (40.6%) periods; however, this difference was not statistically significant (*p* = 0.412). Survival to hospital discharge was lowest during the pandemic (24.5%), whereas identical survival rates were observed in the pre-pandemic and post-pandemic periods (33.3% each), without a statistically significant difference (*p* = 0.530). Similarly, overall mortality was highest during the pandemic (97.3%), although the difference among the study periods did not reach statistical significance (*p* = 0.079). Neurological outcomes among hospital survivors were comparable across the three study periods (*p* = 0.331) (Table 3).

### 3.4. Multivariable Logistic Regression Analysis of Independent Predictors of ROSC and Overall Mortality

To identify independent predictors of return of spontaneous circulation (ROSC), univariable and multivariable logistic regression analyses were performed. The initial model included age, sex, EMS response time, initial cardiac rhythm, initiation of CPR, provider initiating CPR, administration of adrenaline, atropine, and amiodarone, as well as the study period (pre-pandemic, pandemic, and post-pandemic).

Univariable logistic regression identified study period, age, EMS response time, initial cardiac rhythm, administration of adrenaline, and administration of atropine as significant predictors of ROSC. These variables were subsequently entered into the multivariable logistic regression model together with interaction terms between the study period and each significant predictor.

The final model (Table 4) identified EMS response time, initial cardiac rhythm, and the administration of adrenaline and atropine as independent predictors of ROSC. No statistically significant interactions were observed between the study period and any of the significant predictors of ROSC, indicating that the effects of these variables remained consistent across the pre-pandemic, pandemic, and post-pandemic periods.

For each one-minute increase in EMS response time, the odds of achieving ROSC decreased by approximately 9.4%. Administration of adrenaline was associated with lower odds of achieving ROSC (OR = 0.085), whereas administration of atropine was associated with approximately 2.6-fold higher odds of ROSC (OR = 2.608). Initial cardiac rhythm was also a significant independent predictor. Compared with asystole as the reference rhythm, patients presenting with pulseless electrical activity (PEA) had approximately 3.2-fold higher odds of achieving ROSC (OR = 3.158), whereas patients presenting with ventricular fibrillation/pulseless ventricular tachycardia (VF/pulseless VT) had approximately six-fold higher odds of achieving ROSC (OR = 5.998).

The Hosmer–Lemeshow goodness-of-fit test demonstrated good model calibration, indicating adequate agreement between the model and the observed data (χ^2^ = 8.459, df = 8, *p* = 0.390). According to the Omnibus test, the overall model was statistically significant (*p* < 0.001). The Nagelkerke R^2^ value of 0.225 indicated that the model explained approximately 22.5% of the variance in ROSC outcomes.

In the multivariable logistic regression model including interaction terms, age and initial cardiac rhythm remained significant independent predictors of overall mortality, whereas no statistically significant interactions were observed between the study period and these predictors. Increasing age was independently associated with higher odds of mortality (OR = 1.024), corresponding to an approximately 2.4% increase in the odds of death for each additional year of age.

Initial cardiac rhythm was also identified as a strong independent prognostic factor for mortality. Compared with asystole as the reference rhythm, patients presenting with pulseless electrical activity (PEA) had significantly lower odds of mortality (OR = 0.224), indicating a more favorable prognosis. Likewise, patients presenting with ventricular fibrillation/pulseless ventricular tachycardia (VF/pulseless VT) had markedly lower odds of mortality than those presenting with asystole (OR = 0.022), indicating a substantially more favorable prognosis (Table 5).

Overall mortality was independently associated with age and initial cardiac rhythm. The model demonstrated good calibration, as indicated by the Hosmer–Lemeshow goodness-of-fit test (χ^2^ = 2.570, df = 8, *p* = 0.958), indicating good agreement between the predicted and observed outcomes. According to the Omnibus test, the overall model was statistically significant (*p* < 0.001). The Nagelkerke R^2^ value of 0.220 indicated that the model explained approximately 22% of the variance in overall mortality.

The main effect of the study period (pre-pandemic, pandemic, and post-pandemic) was assessed in the multivariable models for both ROSC and all-cause mortality. Although study period was significantly associated with both outcomes in the univariable analyses, these associations were attenuated and no longer statistically significant after adjustment for other clinical predictors. Consequently, study period was not retained in the final multivariable models.

## 4. Discussion

The present study demonstrated significant temporal variation in the incidence of out-of-hospital cardiac arrest (OHCA), with the highest incidence observed during the COVID-19 pandemic and the lowest incidence in the post-pandemic period, although the latter remained comparable to the pre-pandemic period. These findings are consistent with previous studies reporting a significant increase in the incidence of OHCA during the pandemic [10,11]. The largest increase was reported by Nickles et al., who observed a 59.6% rise in OHCA incidence [12]. In contrast, a smaller number of studies have reported either no significant change in OHCA incidence [13,14] or even a reduction in the number of OHCA cases in which EMS teams initiated resuscitation [15]. More recently, Ruiz Azpiazu et al. reported that the increased incidence of OHCA persisted beyond the pandemic period [9].

COVID-19 infection has been shown to cause cardiovascular and pulmonary complications that may increase the risk of OHCA not only during the acute phase of infection but also for days or weeks thereafter [16,17]. However, only approximately 5% of patients with OHCA have evidence of acute COVID-19 infection, suggesting that direct viral infection contributed only modestly to the observed increase in OHCA incidence. Therefore, the observed increase in OHCA incidence during the pandemic was likely driven predominantly by indirect consequences of the pandemic rather than by acute SARS-CoV-2 infection itself, including disruption of cardiovascular prevention programs [18], delayed utilization of EMS and emergency department services due to fear of infection, and reduced access to emergency healthcare [19,20,21]. This interpretation is further supported by reports demonstrating a reduction in the number of patients presenting with ST-segment elevation myocardial infarction during the pandemic [22,23,24]. Delayed treatment of acute myocardial ischemia may facilitate progression to malignant ventricular arrhythmias and subsequent cardiac arrest [25].

In our cohort, the incidence of EMS treated OHCA increased by 16.7% during the pandemic and subsequently declined by 23.7% in the post-pandemic period. The 8.4% lower incidence observed after the pandemic compared with the pre-pandemic period may reflect improvements in secondary cardiovascular prevention and more intensive follow-up of high-risk patients after the pandemic, although this hypothesis warrants further investigation.

All cardiac arrests included in the present study were witnessed, thereby eliminating unwitnessed arrest as a potential confounding factor and strengthening the interpretation of outcomes within the chain of survival. Nevertheless, bystander CPR remained exceptionally uncommon throughout all study periods. The overall proportion of CPR initiated by either EMS personnel or bystanders remained low and relatively stable (19.5%, 17.2%, and 19.8% during the pre-pandemic, pandemic, and post-pandemic periods, respectively), with only a transient decline during the pandemic. Bystander CPR rates were particularly low, accounting for only 2.2%, 1.6%, and 3.0% of cases across the three study periods. Although EMS response times decreased during and after the pandemic, the persistently low contribution of bystander CPR indicates a consistently weak first link in the chain of survival. These findings suggest that the burden of early resuscitation in our region continues to fall predominantly on EMS teams.

Fear of SARS-CoV-2 transmission reduced the willingness of bystanders to initiate CPR before the arrival of emergency medical service (EMS) teams worldwide. Similarly, EMS dispatchers were less likely to instruct bystanders to initiate CPR, particularly in cases of suspected COVID-19 infection [26,27,28,29,30]. Before the pandemic, bystander CPR rates ranged from 25% to 73.5% across different countries [10,31,32]. In the region where the present study was conducted, the corresponding rate was 11.6% in 2010 [33] but had declined to 4.2% immediately before the pandemic [6]. During the pandemic, bystander CPR rates decreased in most countries, ranging from 18.2% to 70% [10,31,32], whereas in some municipalities within our region they declined to as low as 1.7% [6], remaining substantially below the reported European and global averages.

The EuReCa study conducted in this region between 2014 and 2019 showed that the prehospital rate of achieving ROSC and maintaining it until hospital admission was low in the Republic of Serbia, at 8.8% [34]. However, this study was conducted across 40 municipalities whose EMS systems differed organizationally from that in our region, including fewer EMS teams, greater geographical dispersion, and the absence of a dispatch center. Although this rate was higher in our region, it remained substantially below the global and European averages. The decline in survival to hospital admission following EMS-treated OHCA during the pre-pandemic period compared with 2010 may be explained by the intensive training provided to relatives of patients treated at IKVBV in 2009 as part of the “My Heart in Your Hands” project. Consequently, the rate of bystander-initiated CPR during that period was 11.6% [33]. However, the project was subsequently discontinued, resulting in a decline in bystander CPR and, concurrently, in survival to hospital admission among patients with OHCA. The rate of dispatcher-assisted CPR in our region is also low (5.4%), which further contributes to the low rate of bystander CPR and, consequently, to the low rate of survival to hospital admission among patients with OHCA [35].

EMS response times did not differ across the analyzed periods. At the beginning of the pandemic, personal protective equipment was in limited supply; therefore, EMS teams wore full protective suits only when responding to patients with confirmed COVID-19, which was uncommon among highest-priority emergency calls. As the pandemic progressed, it became standard practice not to wear full protective suits when responding to highest-priority calls, with EMS personnel using only masks and gloves as personal protective equipment. Conversely, traffic congestion was substantially reduced during the initial months of the pandemic because of lockdown measures. Collectively, these factors contributed to the absence of a substantial increase in EMS response times during the pandemic.

Kim et al. analyzed 48 studies evaluating OHCA during the COVID-19 pandemic and found a significant reduction in prehospital ROSC rates in 33 of them. This decline was particularly pronounced in Europe and the United States but was not consistently observed in Asia [36]. One study reported a reduction in the ROSC rate from 32.2% to 18.8% during the pandemic in regions with a high incidence of COVID-19 infection [37]. Likewise, the comprehensive analysis by Baldi et al. demonstrated a 73% increase in the proportion of patients who died at the scene and a 32% reduction in survival to hospital admission during the pandemic. Notably, poorer OHCA outcomes were observed regardless of the regional incidence of COVID-19 infection or previously established prognostic factors. Even regions only marginally affected by COVID-19 experienced lower survival to both hospital admission and hospital discharge [10]. Although the mechanisms underlying these observations remain incompletely understood, delayed EMS response times and changes in prehospital resuscitation practices are considered important contributing factors [38].

Consistent with these observations, our region has historically reported relatively low survival to hospital admission following OHCA. Survival to hospital admission was 19.0% in 2010 [33], 18.4% before the pandemic, and declined to 12.6% during the pandemic [6]. Similarly, in the present study, the ROSC rate decreased by 5.2 percentage points during the pandemic compared with the pre-pandemic period and subsequently increased to levels exceeding those observed before the pandemic. Comparable findings were reported by Ruiz Azpiazu et al., who observed an 11.7 percentage-point decline in ROSC during the pandemic, followed by recovery to 30.4% in the post-pandemic period. However, in their study, post-pandemic ROSC rates remained below pre-pandemic values, whereas in our cohort they exceeded pre-pandemic levels. It should also be emphasized that ROSC rates reported by Ruiz Azpiazu et al. were nearly twice as high as those observed in our study across all study periods [9].

Survival to hospital discharge among patients with EMS-treated OHCA was also low across all three study periods (5.4% vs. 2.7% vs. 5.6%) and was substantially lower than that reported in developed countries. However, the EuReCa TWO study reported a survival-to-discharge rate of 8% among patients with OHCA [39], whereas EuReCa THREE reported a rate of 7.5% [40]. These rates were not substantially higher than those observed in our study during the pre-pandemic and post-pandemic periods, whereas survival was substantially lower in our cohort during the pandemic period.

Although the ROSC rate was lowest and overall mortality highest during the pandemic period, the present study demonstrated that both ROSC and overall mortality were primarily determined by clinical factors. Multivariable regression analysis identified EMS response time, initial cardiac rhythm (PEA and VF/pulseless VT), and the administration of adrenaline and atropine as independent predictors of ROSC, whereas age and initial cardiac rhythm were independent predictors of overall mortality. After adjustment for these variables, the COVID-19 pandemic period was not independently associated with either ROSC or overall mortality. Furthermore, no significant interactions were observed between the study period and the major prognostic factors, indicating that their effects remained consistent across the pre-pandemic, pandemic, and post-pandemic periods.

Shorter EMS response times were independently associated with a greater likelihood of achieving ROSC. In addition to EMS response time, initial cardiac rhythm was one of the strongest predictors of outcome, with patients presenting with the shockable rhythm of ventricular fibrillation/pulseless ventricular tachycardia (VF/pulseless VT) having approximately six-fold higher odds of achieving ROSC than those presenting with asystole. Administration of adrenaline was associated with lower odds of ROSC, whereas atropine administration was associated with higher odds. However, these associations should be interpreted in the context of treatment selection and confounding by indication rather than as evidence of causal effects. Adrenaline was administered to nearly all patients except those with shockable rhythms who achieved ROSC after up to three defibrillation shocks, a subgroup with the highest ROSC rate. Conversely, atropine was administered infrequently, predominantly to younger patients with persistent pulseless electrical activity (PEA), which may partly explain its apparent association with improved outcomes.

One of the principal findings of this study is the absence of an independent effect of the COVID-19 pandemic on outcomes after adjustment for established clinical predictors. Although previous studies consistently reported poorer outcomes during the pandemic, particularly during its early phases, these findings have largely been attributed to organizational factors, including prolonged EMS response times, reduced healthcare resource availability, and changes in bystander behavior [41]. Our findings suggest that the pandemic itself did not alter the determinants of survival among patients with EMS-treated OHCA. One of the key findings of this study was that, although the COVID-19 pandemic period was associated with outcomes in the unadjusted analyses, it did not retain independent predictive value after multivariable adjustment. This finding suggests that the pandemic period itself was unlikely to represent an independent risk factor for ROSC or overall mortality after accounting for relevant clinical predictors. However, this result should be interpreted with caution, as some of the variables included in the models may have acted as mediators through which the pandemic exerted its effects, potentially resulting in model over adjustment [42,43]. Furthermore, the influence of unmeasured factors that may have contributed to differences across the study periods—including CPR quality, resuscitation duration, post-resuscitation care, and other organizational characteristics of the healthcare system cannot be excluded [44,45].

Another important finding was the absence of significant interactions between the study period and the major predictors of ROSC or overall mortality, indicating that their prognostic value remained stable over time. This suggests that the fundamental mechanisms determining successful resuscitation remained unchanged despite the substantial organizational challenges imposed by the COVID-19 pandemic, consistent with observations from large OHCA registries [46].

This study has several limitations. First, the retrospective observational design is inherently susceptible to residual confounding. Data were collected from prehospital EMS records, which were not fully compliant with the Utstein Reporting Guidelines. Unfortunately, our institution does not use standardized Utstein reporting templates for OHCA; therefore, we were unable to collect all recommended data elements. Information on comorbidities and patients’ health status before EMS-treated OHCA was unavailable. In addition, data on CPR duration until ROSC, the quality of bystander CPR, the quality of ALS provided by EMS teams, and post-resuscitation care were unavailable, although these factors may substantially influence patient outcomes.

Among patients who did not achieve ROSC, the cause of cardiac arrest could not be determined because this information is not documented in prehospital EMS records. As these patients constituted the majority of the study population, we were unable to analyze the associations of cardiac arrest etiology and reversible causes with ROSC.

An additional limitation of this study is the limited explanatory power of the multivariable models, with Nagelkerke R^2^ values of 0.225 for ROSC and 0.220 for overall mortality, indicating that a proportion of the variability in outcomes remained unexplained by the included predictors. This finding may reflect both the complexity of outcomes following OHCA and the presence of numerous factors that were unavailable for analysis. Although key clinical variables were included in the models, additional factors including CPR duration, details of post-resuscitation care, comorbidities, location of cardiac arrest, and etiology might have further contributed to explaining the observed outcomes. Future studies involving more detailed data collection on patient characteristics, resuscitation quality, and organizational characteristics of the healthcare system may enable more accurate outcome modelling.

## 5. Conclusions

During the COVID-19 pandemic, the incidence of witnessed OHCA) requiring resuscitation by EMS teams increased significantly compared with both the pre-pandemic and post-pandemic periods. In the post-pandemic period, the incidence of EMS treated OHCA decreased to a level lower than that observed before the pandemic.

Patients who experienced EMS treated OHCA during the COVID-19 pandemic had significantly lower rates of ROSC than those in the pre-pandemic and post-pandemic periods. ROSC was achieved significantly more frequently before the pandemic, whereas in the post-pandemic period the ROSC rate did not fully return to the pre-pandemic level. Survival to hospital discharge was also lowest during the pandemic and highest in the post-pandemic period. However, multivariable regression analysis demonstrated that OHCA outcomes were primarily determined by timely and appropriate resuscitation measures. After adjustment for independent clinical predictors, the COVID-19 pandemic was not independently associated with either ROSC or overall mortality. Our findings suggest that the association between the COVID-19 pandemic and outcomes following EMS-treated OHCA may have been mediated by clinical factors. The pandemic itself did not appear to independently alter the established determinants of survival following EMS-treated OHCA. Prospective studies incorporating more detailed data on patient characteristics, the quality of resuscitation, and organizational characteristics of the healthcare system may enable more accurate outcome modeling.

## Figures and Tables

**Table 1:** Demographic and prehospital data patients with OHCA.

	Pre-Pandemic	Pandemic	Post-Pandemic	*p*-Value
Witnessed OHCA ^, *n* (%)	369 (32.1%)	443 (38.5%)	338 (29.4%)	**0.001**
Sex ^‡^				0.733
Male ^, *n* (%)	253 (68.6%)	294 (66.4%)	232 (68.6%)	
Female ^, *n* (%)	116 (31.4%)	149 (33.6%)	106 (31.4%)	
Age (years) ^#^, Mean ± SD	67.1 ± 14.0	67.0 ± 14.5	68.30 ± 14.5	0.405
Time to EMS arrival (min) ^&^, median (Q1–Q3)	7 (5–9)	6 (5–9)	6 (5–9)	0.118
Min–Max	1–23	1–24	1–30	
Immediate CPR attempt ^‡^, Yes (%)	72 (19.5%)	76 (17.2%)	67 (19.8%)	0.567
Immediate CPR attempt ^‡^ by EMS, Yes (%)	58 (15.7%)	62 (14.0%)	49 (14.5%)	0.416
Immediate CPR attempt ^‡^ by haeltcare workers, Yes (%)	6 (1.6%)	7 (1.6%)	8 (2.4%)	0.758
Immediate CPR attempted by bystanders ^‡^, Yes (%)	8 (2.2%)	7 (1.6%)	10 (3.0%)	0.425

^‡^ Chi-square est of independence; ^ χ^2^ Chi-square goodness-of-fit test; ^#^ ANOVA-analysis of variance, post hoc comparisons independent-samples *t*-test; SD: Standard deviation; ^&^ Kruskal-Wallis test, post hoc pairwise comparisons performed using the Mann-Whitney U test; Q1: 25%: Q3: 75%; Min: minimal value; Max: maximal value; OHCA: Out-of-hospital cardiac arrest; EMS: Emergency medical services; CPR: cardiopulmonary resuscitation. Numbers in boldface are statistically significant values.

**Table 2:** Initial Cardiac Rhythm and Advanced Life Support Interventions Performed by EMS Teams.

	Pre-Pandemic ^a^ (*n* = 369)	Pandemic ^b^ (*n* = 443)	Post-Pandemic ^c^ (*n* = 338)	*p*-Value
Procedural interventions:				
Initial rhythm ^‡^				0.480
Asystole ^‡^, Yes (%)	185 (50.1%)	245 (55.3%)	177 (52.4%)	0.334
PEA ^‡^, Yes (%)	117 (31.7%)	115 (26%)	100 (29.6%)	0.187
VF/pulseless VT ^‡^, Yes (%)	67 (18.2%)	83 (18.7%)	61 (18.0%)	0.964
Airway management ^†^				**˂0.001**
Endotracheal intubation ^‡^, Yes (%)	322 (87.3%) ^b^	336 (75.8%)	275 (81.4%) ^b^	**˂0.001**
Bag-valve-mask ventilation ^‡^, Yes (%)	39 (10.6%)	106 (23.9%) ^a^	60 (17.8%) ^a^	**˂0.001**
Laryngeal mask, Yes (%)	1 (0.3)	0 (0.0)	0 (0.0)	0 (0.0)
No airway intervention required, *n* (%)	7 (1.9%)	1 (0.2%)	3 (0.9%)	
Defibrillation (DC shock) ^‡^, Yes (%)	97 (26.3%)	103 (23.3%)	85 (25.1%)	0.597
Number of DC shocks ^&^, median (Q1–Q3)	3 (2–4)	3 (2–4)	2 (1–4)	0.758
Pharmacological interventions:				
Adrenaline administration ^‡^, Yes (%)	357 (96.7%)	429 (96.8%)	326 (96.4%)	0.953
Number of adrenaline ampoules ^&^, median (Q1–Q3)	4 (3–6)	4 (3–6)	4 (2–6)	0.413
Atropine administration ^‡^, Yes (%)	60 (16.3%)	49 (11.1%)	39 (11.5%)	0.061
Number of atropine ampoules ^&^, median (Q1–Q3)	2 (1–2)	3(1–3) ^a^	2 (1–3)	**0.016**
Amiodarone administration ^‡^, Yes (%)	62 (16.8%) ^b,c^	45 (10.2%)	34 (10.1%)	**0.005**
Number of amiodarone ampoules ^&^, median (Q1–Q3)	2 (1–2)	2 (1–2)	2 (1–2)	0.767
Isotonic crystalloid solutions ^‡^, Yes (%)	93 (25.2%) ^b^	80 (18.1%)	92 (27.2%) ^b^	**0.005**

^‡^ Chi-square test of independence, Post hoc comparisons using Bonferroni correction (ꭤ = 0.017); ^†^ Monte Carlo simulation; ^&^ Kruskal-Wallis test, post hoc pairwise comparisons performed using the Mann-Whitney U test; Q1: 25%: Q3: 75%; EMS: Emergency medical services; PEA: Pulseless electrical activity; VF/pulseless VT: Ventricular fibrillation/pulseless ventricular tachycardia; DC shock: Direct current shock. **Note:** Superscript letters (a, b, c) indicate statistically significant differences identified in post hoc analyses. Statistically significant values are presented in **bold**.

**Table 3:** Patient Outcomes and Neurological Status at Hospital Discharge Following EMS-treated OHCA, *n* (%).

	Pre-Pandemic ^a^ (*n* = 369)	Pandemic ^b^ (*n* = 443)	Post-Pandemic ^c^ (*n* = 338)	Total (*n* = 1150)	*p*-Value
ROSC ^‡^					**0.035**
Yes	60 (16.3%) ^b^	49 (11.1%)	57 (16.9%) ^b^	166 (14.4%)	
No	309 (83.7%)	394 (88.9%)	281 (83.1%)	984 (85.6%)	
Admission to the emergency department, Yes	60 (36.1%)	49 (29.5%)	57 (34.3%)	166 (100%)	
Mortality within the first 24 h					0.908
Yes	24 (40%)	21 (42.9%)	25 (43.9%)	70 (42.2%)	
No	36 (60%)	28 (57.1%)	32 (56.1%)	96 (57.8%)	
Mortality within two days or until diacharge					0.412
Yes	16 (44.4%)	16 (57.1%)	13 (40.6%)	45 (46.9%)	
No	20 (55.6%)	12 (42.9%)	19 (59.4%)	51 (53.1%)	
Survival to hospital discharge					0.530
Survived to hospital discharge	20 (33.3%)	12 (24.5%)	19 (33.3%)	51 (30.7%)	
Did not survive to hospital discharge	40 (66.7%)	37 (75.5%)	38 (66.7%)	115 (69.3%)	
Overall mortality					0.079
Yes	349 (94.6%)	431 (97.3%)	319 (94.4%)	1099 (95.6%)	
No	20 (5.4%)	12 (2.7%)	19 (5.6%)	51 (4.4%)	
Neurological status among survivors ^‡^, (*n* = 51)					0.331
No neurological deficit CPC 1	16 (80%)	8 (66.7%)	14 (73.7%)	38 (74.5%)	
Mild neurological deficit CPC 2	3 (15%)	0 (0%)	2 (10.5%)	5 (9.8%)	
Moderate neurological deficit CPC 3	1 (5%)	2 (16.2%)	2 (10.5%)	5 (9.8%)	
Severe neurological deficit CPC 4	0 (0%)	2 (16.2%)	1 (5.3%)	3 (5.9%)	

^‡^ χ^2^ Chi-square test of independence; ROSC: Return of spontaneous circulation. **Note:** Superscript letters (a, b, c) indicate statistically significant differences identified in post hoc analyses. Statistically significant values are presented in **bold**.

**Table 4:** Multivariable Logistic Regression Analysis of Independent Predictors of Return of Spontaneous Circulation (ROSC).

	*p*-Value	OR	95% CI
EMS response time (min) Initial cardiac rhythm (reference: asystole)	**0.012**	0.906	0.839–0.978
Pulseless electrical activity (PEA)	**<0.001**	3.158	1.815–5.496
Ventricular fibrillation/pulseless ventricular tachycardia (VF/pulseless VT)	**<0.001**	5.998	2.764–7.016
Adrenaline administration	**<0.001**	0.085	0.033–0.218
Atropine administration	**0.008**	2.608	1.286–5.287
Study period × EMS response time	0.592	1.016	0.959–1.076
Study period × initial cardiac rhythm	0.722	0.947	0.702–1.278
Study period × adrenaline administration	0.592	1.134	0.717–1.793
Study period × atropine administration	0.343	0.753	0.418–1.354
Constant	0.932	0.954	

OR: Odds ratio; CI: Confidence interval; **Note:** Statistically significant values are presented in **bold**.

**Table 5:** Multivariable Logistic Regression Analysis of Independent Predictors of Overall Mortality.

	*p*	OR	95% CI
Age (years) Initial cardiac rhythm (reference: asystole)	**0.043**	1.024	1.001–1.049
Pulseless electrical activity (PEA)	**0.033**	0.224	0.056–0.889
Ventricular fibrillation/pulseless ventricular tachycardia (VF/pulseless VT)	**<0.001**	0.022	0.004–0.121
Study period × age	0.662	1.002	0.994–1.009
Study period × initial cardiac rhythm	0.182	0.683	0.390–1.196
Constant	0.000	4.463	

OR: Odds ratio; CI: Confidence interval. **Note:** Statistically significant values are presented in **bold**.

## Data Availability

The data presented in this study are available on request from the corresponding author with a reasonable reason.
